# Estimating Short- and Long-Term Associations Between Air Quality Index and COVID-19 Transmission: Evidence From 257 Chinese Cities

**DOI:** 10.3389/ijph.2021.1604215

**Published:** 2021-07-21

**Authors:** Ru Cao, Yuxin Wang, Xiaochuan Pan, Xiaobin Jin, Jing Huang, Guoxing Li

**Affiliations:** Department of Occupational and Environmental Health Sciences, School of Public Health, Peking University, Beijing, China

**Keywords:** COVID-19, transmission, short-term exposure, long-term exposure, air quality index, human mobility

## Abstract

**Objectives:** To evaluate the long- and short-term effects of air pollution on COVID-19 transmission simultaneously, especially in high air pollution level countries.

**Methods:** Quasi-Poisson regression was applied to estimate the association between exposure to air pollution and daily new confirmed cases of COVID-19, with mutual adjustment for long- and short-term air quality index (AQI). The independent effects were also estimated and compared. We further assessed the modification effect of within-city migration (WM) index to the associations.

**Results:** We found a significant 1.61% (95%CI: 0.51%, 2.72%) and 0.35% (95%CI: 0.24%, 0.46%) increase in daily confirmed cases per 1 unit increase in long- and short-term AQI. Higher estimates were observed for long-term impact. The stratifying result showed that the association was significant when the within-city migration index was low. A 1.25% (95%CI: 0.0.04%, 2.47%) and 0.41% (95%CI: 0.30%, 0.52%) increase for long- and short-term effect respectively in low within-city migration index was observed.

**Conclusions:** There existed positive associations between long- and short-term AQI and COVID-19 transmission, and within-city migration index modified the association. Our findings will be of strategic significance for long-run COVID-19 control.

## Introduction

COVID-19, firstly emerging in Wuhan, China has become the focus of global public attention [1, 2]. The epidemic scale of COVID-19 has increased with a rapid increase with confirmed cases increasing across China and worldwide [3]. A mandatory lockdown policy initially launched in Wuhan on January 23 and was followed shortly afterwards by another 95 cities [4]. However, although local government had utilized restriction measures, the coronavirus still spread widely across China. Previous studies have concluded that the spatial distribution of coronavirus infection may not be explained through the epidemic model, geographical distance, population distribution, and age composition [5–8]. Therefore, it will be of great significance to clarify the possible influencing factors such as atmospheric conditions for the COVID-19 epidemic, considering what should be done further to control COVID-19 in the long run [9, 10].

Among the atmospheric conditions, prior studies have explored the impact of ambient air pollution exposure on survival and transmission of coronaviruses. There appeared evidence from China, America, Korea, and Italy [11–14]. However, most of the studies focused on single pollutants, which lacked representativeness of overall air quality conditions and some of them obtained contradictory results [10, 13]. Air quality index (AQI) is a comprehensive indicator reflecting the emissions of particulate matters and gaseous pollutants [15], which may exert a significant impact on the transmission and infection of COVID-19 [16]. This is owing to the fact that COVID-19 is a respiratory disease, and a denser level of adverse air quality exposure would carry the coronavirus in the air for longer, and across larger distances [10]. Also, heavy air pollution could damage lung function and render people more vulnerable to coronavirus disease [17]. Existing literature which employed AQI provided evidence from China that the effect of AQI on confirmed cases associated with an increase in each unit of AQI with statistical significance in several cities [3, 8, 18].

Taking the time scale into consideration, many researchers have demonstrated that the increase of disease incidence or case fatality rate of coronavirus infection was associated with air pollution, with short-term or long-term effects examined separately. For example, Chen, Zhang, and Peng reported the dependent short-term impact of air pollution on COVID-19 transmission [8, 19, 20]; and Yao revealed the long-term effect solely [16]. Early application of the combined method with mutual adjustment in Beijing, China indicated that the mortality effects of short- and long-term exposure to air pollution were related [21]. However, whether the short-term effect affects the daily new confirmed cases of COVID-19 independently from the long-term exposure is still not clear now, as there has been no concerning research. Moreover, scholars pointed out that differences in the structure of fitting models challenge their mutual adjustment in the exposure-response relationship models [21]. Thus, it is imperative to investigate whether and how short-term and long-term exposure to adverse air quality affect the transmission of COVID-19 at the same time.

Hence, our study aimed to provide evidence on AQI and COVID-19 transmission based on data from 257 Chinese cities. A mutually adjusted model was developed to estimate the short-term and long-term effects of AQI simultaneously. The independent effects were also estimated and comparisons were made to the previous model to help us better understand the associations. We also explored the effect modification of within-city migration index.

## Methods

### Study Design and Data Collection

Our research included 257 cities in mainland China between January 23 to April 30, 2020. At least one confirmed case was reported for each of the sample cities. The beginning point was set after January 23 when Wuhan locked down. Cities from Hubei were excluded to eliminate the effects of extreme values and endogenous influence. Cities from Qinghai province and Tibet were also excluded as the local confirmed cases were mainly imported cases from abroad. Plus, cities lacking air pollution data were excluded to confirm the quality of database.

Daily new confirmed cases for each city were selected as the dependent variables in our study and gathered from daily reports from the China National Health Commission and the Provincial Health Commissions. Artificial distortion occurred in Hubei Province on February 12, 2020 and in other provinces on February 20, 2020 due to changes in diagnostic criteria. To deal with this artificial distortion, we used a 5-days moving average of the number of confirmed COVID-19 cases to replace case number on the day [22]. Air pollution, measured by the air quality index (AQI) was regarded as the crucial independent variable. Hourly data of AQI were downloaded from the National Urban Air Quality Publishing Platform, a platform administered by China’s Ministry of Environmental Protection. The individual air quality index (IAQI) values for six pollutants (PM_2.5_, PM_10_, NO_2_, O_3_, SO_2_, and CO) were calculated by comparing each pollutant to its standard, and the maximum of the six IAQIs was reported as AQI [23]. The details could be found in Supplementary Table S1 of supplemental materials.

With respect to the covariates, first we obtained daily meteorological data on mean temperature, relative humidity, and wind speed from the National Meteorological Information Center. Second, the city-level covariates were retrieved from China City Statistical Yearbook 2019. Third, considering the huge impact of human mobility, the real-time intracity migration index was calculated from the ratio of the number of people traveling in a city to the number of people living in that city [24]. Immigration index and within-city migration index was attained from the Baidu Qianxi online platform, which collected human movements through tracking mobile phone data from Baidu location-based services. Fourth, we also controlled the logarithm of the number of new confirmed cases in city i and the whole country on day t–1 to account for potential serial correlation and the effects of national intervention policy on COVID-19 in China [8, 25]. Additionally, the introduction of province in the regression model aimed to control the influence of provincial spatial characteristics. The missing meteorological data (<1%) were interpolated by imputing with the mean of the previous day and the next day.

### Statistical Analysis

We fit a Quasi-Poisson regression based on generalized additive model (GAM) to estimate the association between air pollution exposure and COVID-19 daily new confirmed cases. The quasi-Poisson model was used to control for possible overdispersion [26]. To investigate the lag pattern of AQI, potentially delayed and cumulative associations were estimated in multiple lag days within 1 week (mv0∼0, mv0∼1, mv0∼2, mv0∼3, mv0∼4, mv0∼5, mv0∼6). Comparing the percent change (%) in daily new confirmed cases estimated by the model with both short- and long-term exposure to air pollution at all lag days, the 4-days moving average (mv0∼3) of AQI showed the largest estimate with statistical significance, AQI at mv0∼3 was selected in our main analysis.

$$\text{log}\left(\mu_{\text{it}}\right)=\lambda_{\text{i}}+\beta_{1}{\text{×AQI}}_{\text{long}-\text{term}}+\beta_{2}{\text{×AQI}}_{\text{short}-\text{term}}+\beta_{3}\text{×Immigration index }+\beta_{4}\text{×Within}-\text{city migration index}+\beta_{5}\text{×log}\left({\text{Y}}_{\text{i,t}-1}\right)+\beta_{6}\text{×log}\left({\text{Y}}_{\text{country,t}-1}\right)+s\left(\text{meteorological covariates, df}=3\right)+\text{ city}-\text{level covariates}+\text{factor}\left(\text{province}\right)+\text{dow}$$ (1)

Where *μ*_*it*_ is expected confirmed case in city i on day-of-year t; *λ*_*i*_ is a random intercept for each city; *β*_*1*_ and *β*_*2*_ are coefficients of short-term and long-term impact of AQI; *AQI*_*long-term*_ is the 1-year moving average ending on day t in each city; *AQI*_*short-term*_ is the deviation of the 4-days average from its long-term average (AQI_long-term_) in each city; *log(Y*_*i,t-1*_ *)* and *log(Y*_*country,t-1*_ *)* indicate the logarithm of the number of confirmed new cases in city i and the whole country on day t-1, respectively; *s* means the spline function of meteorological covariates with 3 degrees of freedom including mean temperature (°C), relative humidity (%) and wind speed (m/s); and city-level covariates include population density (per 1 km [2]), doctors per 10,000 people; proportion of unemployed (per 10,000 people); proportion of green space and Gross Domestic Product (GDP) per capita (10,000 yuan) data. To control the spatial heterogeneity, province is introduced to the regression model as a categorical variable; *dow* was an indicator variable for “day of week” to account for possible variation in a week.

On the first hand, short-term impact of AQI without mutual adjustment was separately estimated by emitting the long-term impact term in Eq. 1. Long-term impact was also estimated in a similar way. On the other hand, the Quasi-Poisson regression model with an intercept was used to fit daily new confirmed cases with both short- and long-term AQI simultaneously [27]. In this mutually adjusted model, the long-term exposure was subtracted from the short-term exposure to avoid collinearity issues. The potential confounding originated from the spatial difference in AQI was controlled when assessing the short-term effects. Only time-varying variables at a specific location could contribute to the short-term effects in the analysis [21]. In all models, the percent change (%) in COVID-19 transmission corresponding to 1 unit increase in AQI was estimated. We also conducted a stratified analysis using within-city migration index as the stratifying factor to explore the modifying effect of within-city migration index. We first calculated the average within-city migration index in each city during the study period and obtained the median value of 257 cities as the cutoff that was 4.01. Then we could divide the data into two layers for stratified analysis.

Finally, for both short-term and long-term analyses, we fit penalized regression splines in the model to assess the dose-response relationship and capture the shape of curve. All the statistical analyses were conducted using R version 3.6.2. Statistical significance was defined as two-sided *p* < 0.05.

## Results

### Descriptive Statistics

**FIGURE 1:**
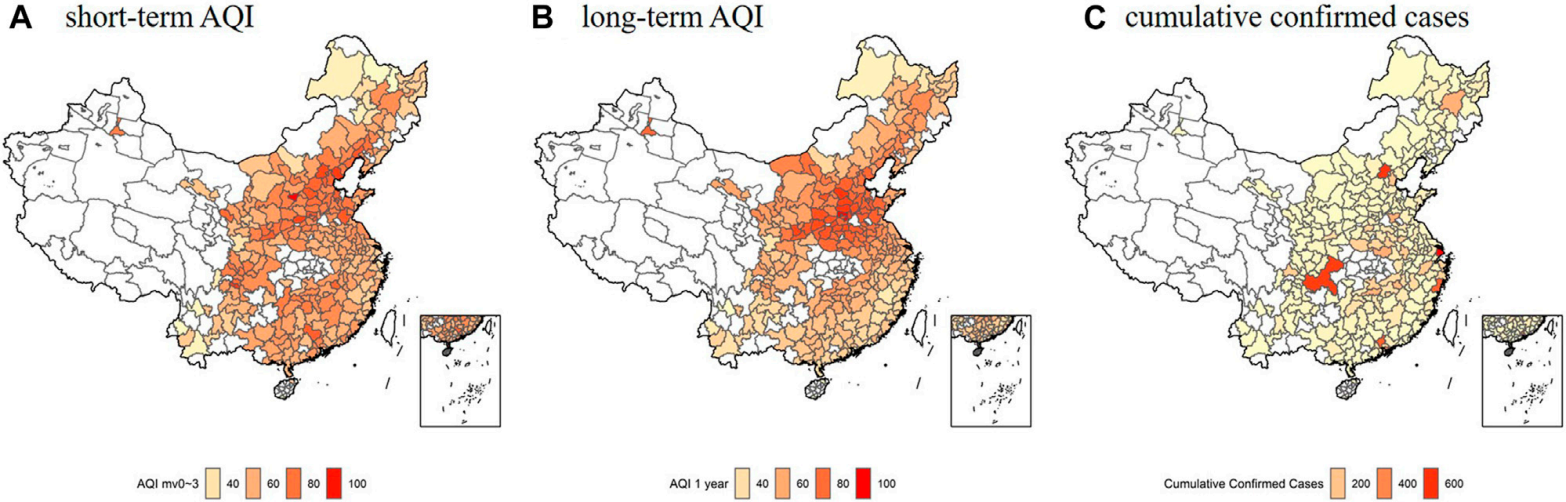
The spatial distribution of COVID-19 cumulative confirmed cases, long- and short-term air quality index in 257 cities in China, 2020. **(A)** The spatial distribution of short-term air quality index (mv0-3) on April 30, 2020. **(B)** The spatial distribution of long-term air quality index (1-year average) on April 30, 2020. **(C)** The cumulative confirmed cases on April 30, 2020. Estimating Short- and Long-Term Associations Between Air Quality Index and COVID-19 Transmission: Evidence From 257 Chinese Cities, China, 2020.

Table 1 presents the descriptive statistics of COVID-19 daily confirmed cases, AQI and other covariates in 257 Chinese cities during January 23 to April 30, 2020. The average of daily confirmed cases, air quality index and within-city migration index were 0.52, 62.04, and 3.97, respectively. AQI ranged from 9 to 451 during the study period and long-term AQI (1 year average) ranged from 27.45 to 110.21. In the study period, PM_2.5_, PM_10_ and O_3_ were responsible for AQI. PM_2.5_, PM_10_ and O_3_ were driving the AQI in 59, 17.5, and 22% of the days, respectively. Average daily mean temperature, relative humidity and wind speed were 9.52°C, 65.21%, and 2.38 m/s, respectively. As for the city-level variables, the average immigration index and within-city migration index were 0.74 and 3.97. The mean population density, proportion of doctors and unemployed were 450.80, 26.21, and 61.67. Average proportion of green space and Gross Domestic Product (GDP) per capita were 0.01 and 6.04. Figure 1 shows the spatial distribution of COVID-19 cumulative confirmed cases, long- and short-term AQI on April 30, 2020 in 257 cities in China. Overall, both short- and long-term AQI were higher in northern China and lower in southern area.

**TABLE 1:** Summary characteristics of COVID-19 daily confirmed cases, meteorological and pollution variables, and covariates from January 23 to April 30, 2020, China. Estimating Short- and Long-Term Associations Between Air Quality Index and COVID-19 Transmission: Evidence From 257 Chinese Cities, China, 2020.

Variables	Mean(SD)	Min	Max
Daily confirmed cases	0.52(2.66)	0.00	201.00
Immigration index	0.74(0.92)	0.01	11.37
Within-city migration index	3.97(1.22)	0.30	8.88
Mean temperature (°C)	9.52(8.44)	−27.90	28.20
Relative humidity (%)	65.21(20.39)	6.33	100.00
Wind speed (m/s)	2.38(1.23)	0.00	15.40
AQI	62.04(35.71)	9.00	451.00
AQI-1 year average	60.95(16.28)	27.45	110.21
Population density (per square kilometer)	450.80(361.33)	9.79	2,578.45
GDP per capita (ten thousand yuan)	6.04(3.45)	1.27	18.96
Proportion of unemployed (per ten thousand people)	61.67(45.03)	4.42	314.11
Doctors per ten thousand people	26.21(12.27)	11.00	88.31
Proportion of green space	0.01(0.04)	0.00	0.49

### Short- and Long-Term Exposure Effects in Both Models With or Without Mutual Adjustment

Table 2 shows the estimated percent change in daily new confirmed cases with 95% CIs for a 1 unit increase for both short-term and long-term AQI with or without mutual adjustment in two models. Model a was adjusted for covariates including meteorological conditions, human mobility, and COVID-19 cases, while model b further controlled the extra city-level covariates. Long- and short-term AQI was significantly associated with COVID-19 transmission in both models. With mutual adjustment, in the fully-adjusted model b, we found a significant 1.61% increase in daily confirmed cases (95%CI: 0.51%, 2.72%) for each 1 unit increase in long-term AQI. A 0.35% increase was observed (*p* < 0.001, 95%CI: 0.24%, 0.46%) in short-term AQI. Without mutual adjustment, a significant 2.21% (95%CI: 1.10%, 3.33%) and 0.37% (95%CI: 0.26%, 0.48%) increase in daily new confirmed cases per 1 unit increase for long-term and short-term AQI in model b, respectively. Both long- and short-term effects increased slightly in model a. The results were relatively robust to the choice of lag pattern for short-term impact.

**TABLE 2:** Percent change in COVID-19 daily new confirmed cases (95% CI) per 1 unit increase for both short-term and long-term air quality index with or without mutual adjustment. Estimating Short- and Long-Term Associations Between Air Quality Index and COVID-19 Transmission: Evidence From 257 Chinese Cities, China, 2020.

AQI	Percent change(%, 95%CI)	*p* value
with mutual adjustment		
short-term AQI^a^	0.35(0.24, 0.46)	<0.001
short-term AQI^b^	0.35(0.24, 0.46)	<0.001
long-term AQI^a^	2.46(1.44,3.48)	<0.001
long-term AQI^b^	1.61(0.51, 2.72)	0.004
without mutual adjustment		
short-term AQI^a^	0.39(0.28, 0.49)	<0.001
short-term AQI^b^	0.37(0.26, 0.48)	<0.001
long-term AQI^a^	3.07(2.05, 4.11)	<0.001
long-term AQI^b^	2.21(1.10, 3.33)	<0.001

a Model a: adjusted for covariates including meteorological conditions: daily average temperature, relative humidity, wind speed; human mobility: immigration index, within-city migration index; Cases: the logarithm of the number of new confirmed cases on day t-1 in each city and the country-level daily new confirmed cases.

b Model b: adjusted for above covariates including extra city-level covariates. City-level covariates: population density (per 1 km [2]), doctors per 10,000 people; proportion of unemployed (per 10,000 people); proportion of green space; GDP per capita (10,000 yuan).

In addition, in the mutually adjusted model, COVID-19 transmission attributed to short- and long-term AQI was smaller than those estimated by the model without mutual adjustment. Daily new confirmed cases due to the long-term effects of AQI were much larger than those caused by short-term effects.

### Exposure-Response Curves for the Association Between Long-Term and Short-Term Air Quality Index and Log Relative Risk With Mutual Adjustment

**FIGURE 2:**
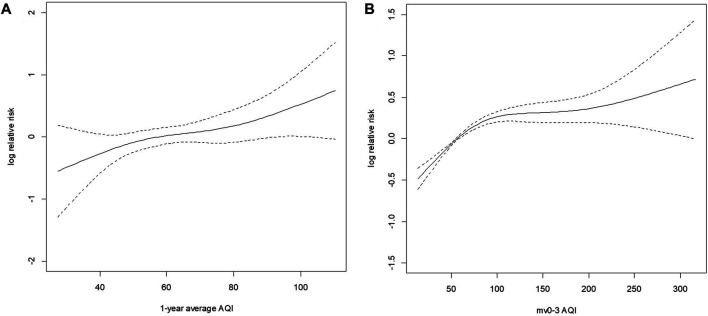
The exposure-response curves for the association between long-term and short-term air quality index and log relative risk with mutual adjustment. **(A)** The exposure-response curves for the association between long-term air quality index and log relative risk with mutual adjustment. **(B)** The exposure response curves for the association between short-term air quality index and log relative risk with mutual adjustment. Estimating Short- and Long-Term Associations Between Air Quality Index and COVID-19 Transmission: Evidence From 257 Chinese Cities, China, 2020.

Figure 2 demonstrates the exposure-response curves for the association between long-term and short-term AQI and log relative risk with mutual adjustment. The curves showed a consistent increasing tendency. We can see a nonlinear relationship between short-term AQI and log relative risk of COVID-19 transmission. The association was linear with a steeper slope when AQI < 100 and the curve appeared to flatten at a higher concentration up to 200 and showed a slow linear increase again. A larger confidence interval was also observed in the high pollution level. As to the long-term impact, the dose-response curve was almost linear with no discernible threshold.

### Short- and Long-Term Exposure Effects in Both Models With Mutual Adjustment Stratified by Within-City Migration Index

On account that since the lockdown policies put into effect, the trip intensity within the city may play the dominant role. As shown in Table 3, for short-term impact, a 0.41% increase (95%CI: 0.30%, 0.52%) in daily new confirmed cased was observed in low within-city migration index stratum and the difference between subgroups were significant (*p* = 0.011), suggesting stronger association between short-term impact of AQI and COVID-19 daily new confirmed cases when the within-city migration index was low. Similarly, a non-significant effect of 2.33% (95%CI: 0.20%, 4.50%) in high within-city migration index stratum compared with low within-city migration index stratum (1.25%, 95%CI: 0.04%, 2.47%) for long-term effect was discovered, and there existed no statistical significance between two subgroups. The findings showed that with regards to both short- and long-term exposure, only when within-city migration index was low could the effects of AQI on COVID-19 transmission be found which supported that cases in high intensity groups may be mainly influenced by population mobility, and then the effects of AQI may be masked.

**TABLE 3:** Percent change in COVID-19 daily new confirmed cases (95% CI) per 1 unit increase for both short-term and long-term air quality index stratified by within-city migration index. Estimating Short- and Long-Term Associations Between Air Quality Index and COVID-19 Transmission: Evidence From 257 Chinese Cities, China, 2020.

AQI	Percent change(%, 95%CI)	*p* value
short-term AQI		
high	0.11(−0.09, 0.31)	0.011
low	0.41(0.30, 0.52)	
long-term AQI		
high	2.33(0.20, 4.50)	0.391
low	1.25(0.04, 2.47)	

^a^ *p* values were obtained from Q test.

^b^high means high within-city migration index.

^c^low means low within-city migration index.

## Discussion

To the best of our knowledge, this is the first multi-city study estimating the associations between short-term and long-term exposure to air pollution and COVID-19 daily new confirmed cases simultaneously. We aimed to give evidence on the impact of air pollution exposure and COVID-19 transmission to control the long-run disease pandemic, especially in high pollution level countries like China. Results showed that the model with mutual adjustment of short-term and long-term impacts provided better estimation of AQI related health effects that has not been employed on COVID-19 transmission previously in China. We concluded that both short- and long-term AQI increased the daily confirmed cases, and their effects were overestimated without the mutual adjustment of them. Plus, long-term exposure to air pollution showed a stronger effect on COVID-19 transmission than short-term exposure.

For long-term effects, our finding revealed a 1.61% (95%CI: 0.51%, 2.72%) increase for each 1 unit increment of AQI. An ecological analysis performed in Canadian health regions reported that long-term PM_2.5_ exposure exhibited a positive association with COVID-19 incidence (RR = 1.07, 95%CI: 0.97, 1.18) [28]. Nevertheless, research from 49 Chinese cities revealed results that every 10 μg/m^3^ increase in 4-years average level PM_2.5_ and PM_10_ concentrations, the COVID-19 CFR increased by 0.61% (95%CI: 0.09%, 1.12%) and 0.33% (95%CI: 0.03%, 0.64%), respectively [16]. Results from our study showed a larger impact of AQI compared with domestic studies. This may be due to the inconsistency of endpoint and study countries in various studies as there was no study on long-term effect on daily new confirmed cases in China so far. For short-term effects, a study in Japan found an increased risk of confirmed cases (RR = 1.03, 95%CI: 1.00, 1.06) associated with PM_2.5_ exposure [29]. Zoran et al. study showed that high levels of urban air pollution have a significant impact on the increased rates of confirmed COVID-19 total number, daily new cases, and total Deaths [30]. Another study conducted in 120 Chinese cities demonstrated that daily confirmed cases increased by 0.16% per 1 unit increase of AQI [18]. These findings held important implications for the interpretation of previous studies without mutual adjustment. Our study observed a 0.37% (95%CI: 0.26%, 0.48%) increase associated with AQI which was similar to prior studies.

Higher estimates were found for both long- and short-term effect without mutual adjustment compared with those with mutual adjustment, consistent with prior study using the same method [21], indicating that long-term effect of air pollution may bias the associations of short-term exposure and daily new confirmed cases in the model without mutual adjustment. Part of the cases during the time period at a specific location could be caused by the long-term exposure of air pollution and thus both short-term and long-term effects of air pollution on COVID-19 transmission may be misestimated in studied with only short- or long-term exposure considered. Through applying such a mutually adjusted model, we managed to estimate both the independent effects on COVID-19 transmission attributable to short- and long-term exposure of air pollution simultaneously. Nevertheless, another similar study conducted in New England reported different results: without mutual adjustment, smaller estimates were found for both short-term and long-term effects of PM_2.5_ exposure to mortality than for those with mutual adjustment [27]. Pollution levels in China and New England variation may lead to the inconsistency.

Although the short-term and long-term effects were partly dependent on each other, we found long-term effect was more pronounced than short-term effect in the overall analysis with or without mutual adjustment. The long-term effect was much larger than the short-term effect after controlling for the short-term exposure, indicating that there existed long-term effects of air pollution which cannot be solely explained by short-term effects. Elevated AQI level, which means heavier exposure to air pollution, may increase the risk of COVID-19 transmission transiently within several days or even hours [31, 32]. When considering the long- and short-term effect separately, the biological mechanism behind the long-term effect may lie in the enhanced susceptibility of population [33]. Prior studies demonstrated greater susceptibility to particulate matter (PM) exposure-induced C reactive protein (CRP) response which could also be partly attributed to the susceptibility to air pollution effects. Furthermore, although the transmission of COVID-19 has not been proved to be related with inflammation and oxidative stress, previous studies have supported that inflammation and oxidative stress do harm to the ability of the immune system and COVID-19 is an infectious disease strongly associated with immunity [34]. The cumulative damage may contribute to a larger impact on the susceptibility to air pollutant-related abnormality of a population faced with infectious disease. While for the short-term, it is possibly due to the corresponding spread of virus, as the potential role of air pollution on viral spread and infectivity has been explored in one first preliminary observational study of consistent association between the number of COVID-19 infected people and PM_10_ peaks [5].

The results of stratified analysis demonstrated the fact that the impact of air pollution exposure on COVID-19 transmission was more announced when the within-city migration index was low. Actually, although the lockdown policy promoted the air quality as a previous study reported, limiting human movements could reduce COVID-19 cases by improving air quality besides decreasing social contact [18], the impact caused by the flow of people may be sufficiently great and therefore mask the impression that daily new confirmed cases were AQI-related, so that the impact of AQI on COVID-19 transmission can only be captured when the within-city migration index was low, which means that the impact of the flow of people was weak. Another possible reason for the non-significant effect for cities with a high within-city migration index was insufficient statistical power.

In this study, we estimated the long- and short-term effect of air pollution on COVID-19 transmission simultaneously, which was our major contribution. Furthermore, we explored the effect of modification of within-city migration index and tried to interpret the potential infection source. The present study also had several limitations. First, the human mobility index data from Baidu Qianxi Platform may not report the real number of people going outdoors, which may result in inaccurate estimates. Second, the study domain limited in mainland China and the air pollution level was relatively high in contrast with other countries which may not represent the whole situation across the world. However, considering the demand in adjusting long-term COVID-19 transmission control policy and strategies in our country, evidence from mainland China is necessary. Third, we measured air pollution by AQI. The disadvantage of AQI is that it does not consider the interaction of various types of pollutants meaning that AQI cannot capture the additive effects of multiple air pollutants [35]. Despite its shortcomings, compared with single types of air pollutants, the AQI is a comprehensive indicator. Besides, the AQI currently reported in many countries and regions around the world is a simple and easily understandable numeric scale ranging from 0 to 500 [36], so we chose AQI as the indicator of air pollution.
