# Supplementary material for: Estimating Short- and Long-Term Associations Between Air Quality Index and COVID-19 Transmission: Evidence From 257 Chinese Cities
Source: Int J Public Health. 2021 Jul 21;66:1604215. doi: 10.3389/ijph.2021.1604215 (PMC8333027; doi:10.3389/ijph.2021.1604215)
Supplement: Supplementary file 1 [file Table1.DOCX]

The AQI is calculated by two steps. In the first step, the individual air quality index (IAQI) values for six pollutants (PM_2.5_, PM_10_, NO_2_, O_3_, SO_2_, and CO) were calculated by combining the average concentration limits of the different pollutants via Eq (1) ^1^. The second step is to choose the maximum of the six IAQI values as the AQI ^2^.

$IAQI=\frac{I_{high}-I_{low}}{C_{high}-C_{low}}\left( C-C_{low} \right)+I_{low}$ (1)

where $C$ is the monitored ambient average concentration of pollutant i; $C_{low}$is the breakpoint lower than or equal to $C$; $C_{high}$ is the breakpoint higher than or equal to $C$; and $I_{low}$ and $I_{high}$ are the IAQI values corresponding to $C_{low}$ and $C_{high}$, respectively.

**Supplementary Table1. Individual air quality index (IAQI) and corresponding pollutants concentration limits in China.**

| **IAQI Values (I)** | $I_{low}$ | 0 | | 51 | | 101 | | 151 | | 201 | | 301 | | 401 | |
| --- | --- | --- | --- | --- | --- | --- | --- | --- | --- | --- | --- | --- | --- | --- | --- |
|  | $I_{high}$ | 50 | | 100 | | 150 | | 200 | | 300 | | 400 | | 500 | |
| **Pollutant concentrations** | | $C_{low}$ | $C_{high}$ | $C_{low}$ | $C_{high}$ | $C_{low}$ | $C_{high}$ | $C_{low}$ | $C_{high}$ | $C_{low}$ | $C_{high}$ | $C_{low}$ | $C_{high}$ | $C_{low}$ | $C_{high}$ |
| PM_2.5_-24 h | (μg/m^3^) | 0 | 35 | 35.1 | 75 | 75.1 | 115 | 115.1 | 150 | 150.1 | 250 | 250.1 | 350 | 350.1 | 500 |
| PM_10_-24 h | (μg/m^3^) | 0 | 50 | 51 | 150 | 151 | 250 | 251 | 350 | 351 | 420 | 421 | 500 | 501 | 600 |
| CO-24 h | (ppm) | 0 | 2 | 2.1 | 4 | 4.1 | 14 | 14.1 | 24 | 24.1 | 36 | 36.1 | 48 | 48.1 | 60 |
| CO-1 h | (ppm) | 0 | 5 | 5.1 | 10 | 10.1 | 35 | 35.1 | 60 | 60.1 | 90 | 90.1 | 120 | 120.1 | 150 |
| SO_2_-1 h | (ppb) | 0 | 150 | 151 | 500 | 501 | 650 | 651 | 800 | - | - | - | - | - | - |
| SO_2_-24 h | (ppb) | 0 | 50 | 51 | 150 | 151 | 475 | 476 | 800 | 801 | 1600 | 1601 | 2100 | 2101 | 2620 |
| O_3_-8 h | (ppb) | 0 | 100 | 101 | 160 | 161 | 215 | 216 | 265 | 266 | 800 | - | - | - | - |
| O_3_-1 h | (ppb) | 0 | 160 | 161 | 200 | 201 | 300 | 301 | 400 | 401 | 800 | 801 | 1000 | 1001 | 1200 |
| NO_2_-24 h | (ppb) | 0 | 40 | 41 | 80 | 81 | 180 | 181 | 280 | 281 | 565 | 567 | 750 | 751 | 940 |
| NO_2_-1 h | (ppb) | 0 | 100 | 101 | 200 | 201 | 700 | 701 | 1200 | 1201 | 2340 | 2341 | 3090 | 3091 | 3840 |

Source: ^1^

PM_2.5_, PM_10_ and O_3_ were responsible for AQI in the study period. PM_2.5_, PM_10_ and O_3_ were driving the AQI in 59%, 17.5% and 22% of the days, respectively.

**References**

1. Tan X, Han L, Zhang X, Zhou W, Li W, Qian Y. A review of current air quality indexes and improvements under the multi-contaminant air pollution exposure. *J Environ Manage*. Feb 1 2021;279:111681. doi:10.1016/j.jenvman.2020.111681

2. Zhu Y, Xie J, Huang F, Cao L. The mediating effect of air quality on the association between human mobility and COVID-19 infection in China. *Environ Res*. Oct 2020;189:109911. doi:10.1016/j.envres.2020.109911
